# Exploring Sexually Transmitted and Bloodborne Infection Rates in Florida After Implementation of Syringe Services Programs: A Quasi-Experimental Study

**DOI:** 10.1016/j.focus.2026.100536

**Published:** 2026-07-22

**Authors:** Bedis Elkamel, Shelby M. Meaders, Megan E. Murphy, Teresa A. Chueng, Hansel E. Tookes, Joy D. Scheidell

**Affiliations:** 1 Department of Health Sciences, College of Health Professions and Sciences, University of Central Florida, Orlando, Florida; 2 Division of Infectious Diseases, Department of Medicine, University of Miami Miller School of Medicine, Miami, Florida

**Keywords:** Harm reduction, sexually transmitted infections, HIV, HCV

## Abstract

**Introduction:**

The aim of this study was to evaluate the associations between syringe services program implementation and sexually transmitted and blood-borne infection rates in Florida counties.

**Methods:**

A difference-in-difference analysis was conducted using surveillance data (2010–2023) to compare sexually transmitted and blood-borne infection rates, including nonviral sexually transmitted infections (i.e., chlamydia, gonorrhea, infectious syphilis), acute hepatitis C virus, and HIV, before and after syringe services program implementation in Florida counties with operational syringe services programs versus control counties without syringe services programs. Two-way fixed-effects models were used to estimate average treatment effects, and multiple sensitivity analyses explored the robustness of the results.

**Results:**

Syringe services program implementation was associated with significant reductions in HIV rates (−11 per 100,000; 95% CI= −15.4, −6.7 in Miami-Dade County vs control counties), and hepatitis C virus rates appeared stable, whereas nonviral sexually transmitted infection rates appeared to increase.

**Conclusions:**

Syringe services programs appear to be effective in reducing HIV at the population level in Florida. The observed increase in sexually transmitted infection rates may reflect improved detection facilitated by syringe services programs and linkage to other care. Findings support the expansion of syringe services programs as a public health strategy and highlight the need for research on their broader population-level impact.

## HIGHLIGHTS

• Syringe services programs (SSPs) are linked to significant population‑level reductions in HIV rates.
• Sexually transmitted infection rate increases may reflect improved screening through SSP services.
• Hepatitis C virus rates remained stable after SSP implementation.
• Findings support SSP expansion to strengthen public health outcomes.

## INTRODUCTION

Sexually transmitted infections (STIs), HIV, and hepatitis C virus (HCV) are prevalent among people who inject drugs (PWID) in the U.S.^1^^,^^2^ Among PWID, blood-borne viral infections such as HIV and HCV may be transmitted through the receptive sharing of syringes and injection equipment.^3^ The risk of nonviral STIs caused by *Chlamydia trachomatis, Neisseria gonorrhea*, and *Treponema pallidum* is elevated among PWID owing to factors such as multiple or concurrent sexual partnerships, survival sex practices including sex work, lack of access to STI screening and treatment, and the high prevalence of untreated STIs in overlapping drug use and sexual networks.^4^^,^^5^ Drug use, including injection drug use, is increasingly reported among people diagnosed with STIs.^6^ Prevention of STIs, HIV, and HCV among PWID remains a significant public health priority.

Beginning with the HIV epidemic in the 1980s, syringe services programs (SSPs) have served as a trusted, low-barrier, and vital resource for harm reduction and preventing the spread of infectious diseases among PWID, who frequently face barriers to engaging with traditional healthcare settings.^7^ Given the direct spread of HIV/HCV through receptive syringe sharing, historically, SSPs have focused on the prevention of these blood-borne pathogens and have shown effectiveness in reducing HIV and HCV.^8^ Research has not focused on the effects of SSPs on nonviral STIs, although, considering that SSPs frequently offer resources and services that may reduce STI transmission, such as condoms, STI testing and screening, and vaccinations,^9^ SSPs may reduce the risk of STIs and related adverse sexual and reproductive health outcomes.^10^

SSPs have been increasingly implemented throughout the U.S., although their legal authorization in Florida is relatively recent.^11^^,^^12^ After legislative changes and a rise in substance use in Miami, FL in the 2010s, medical students evaluated the need for and feasibility of implementing SSPs.^13^ The Miami SSP was launched as a pilot program in December 2016 and has shown sustained success, including naloxone distribution to reduce opioid-related deaths, early HIV detection and viral suppression, decreases in improperly discarded syringes, the ability to serve as treatment hubs for skin and soft tissue infections, and cost-effectiveness in improving health among PWID.^11^^,^^14^, ^15^, ^16^ In 2019, Florida passed the Infectious Disease Elimination Act (IDEA) legislation authorizing counties to opt in to establishing and operating SSPs throughout the state, although, presently, most counties have not yet passed such legislation and implemented operational SSPs. In Florida and throughout the U.S., there continue to be barriers to the widespread implementation of SSPs, including stigma and misunderstanding in the community, political and legal resistance, funding and resource constraints, and logistical and infrastructure challenges.^17^

Although the evidence has demonstrated the benefits of SSPs, gaps in knowledge remain. First, most prior research on the effects of SSPs has focused on reductions in HIV and HCV and has not yet explored potential effects on nonviral STI risk, despite SSPs offering services that may impact these infections.^9^ Second, research often examines the effects of SSPs on individual-level outcomes, and research estimating population-level effects is limited or based on simulation studies.^18^^,^^19^ Finally, no known studies have utilized a difference-in-difference (DID) design to explore the associations between SSPs and both sexually transmitted and blood-borne infection rates; this quasi-experimental approach may provide an improved understanding of the population-level impact of SSPs and offer insights into their effectiveness in broader public health contexts.

## METHODS

This study utilizes surveillance data from the Florida Department of Health to measure incident STIs (i.e., chlamydia, gonorrhea, infectious syphilis [primary and secondary]), acute HCV, and HIV rates, standardized per 100,000 residents from 2010 to 2023.^20^ The Florida Department of Health and the North American Syringe Exchange Network were used to identify Florida counties with and without operational SSPs,^12^ which were used to define the comparison counties and the study periods in the analyses described below. The IRB of the University of Central Florida considers the use of publicly available deidentified data to be nonhuman subjects research that does not require ethical review.

Considering that SSPs in Florida were implemented in different counties at different times after legislation, multiple DID analyses were conducted that varied the definitions of treatment and control groups as well as pre and postimplementation periods to reflect the timing of SSP implementation and assess the robustness of the findings under different assumptions.

Primary analyses used Miami-Dade as the treatment county. IDEA Miami became operational in December 2016, and the most recent year for county-level STI, HIV, and HCV rate data was 2023. Therefore, the pre-SSP implementation period was defined as 2010–2016, and the post-SSP implementation period was defined as 2017–2023. Miami-Dade County was used as the treatment county and compared with control counties that had not yet implemented SSPs during the study period, including Bay, Collier, Pasco, Polk, and St. Lucie Counties. Florida currently has 7 counties that are designated as Ending the HIV Epidemic priority jurisdictions, including Broward, Duval, Hillsborough, Miami-Dade, Orange, Palm Beach, and Pinellas.^21^ The control counties used in the analyses were selected on the basis of the authors’ expert knowledge of SSP implementation and operationalization in Florida.

Additional analyses combined treatment counties. Broward and Palm Beach counties are geographically proximal to Miami-Dade county, but they did not implement their own legal SSPs until 2021. To account for the staggered SSP implementation, additional analyses were conducted in which Miami-Dade, Broward, and Palm Beach were treated collectively as treatment counties. In these models, the pre-SSP implementation period was restricted to 2018–2020, and the post-SSP period was defined as 2021–2023 to ensure that all treatment counties had an operational SSP during the post-SSP implementation period and to align the duration of preimplementation and postimplementation periods.

To account for potential delays in observing population-level effects after SSP implementation, sensitivity analyses were conducted using alternative definitions of pre and postimplementation periods. Specifically, the preimplementation period for Miami-Dade county was restricted to 2010–2015, and the postimplementation period was restricted to 2018–2023 to allow for a transition period after SSP program initiation. Additional sensitivity analyses restricted the observation period for Miami-Dade to shorter balanced timeframes comparable in length to those used in the analyses with Broward and Palm Beach counties.

To understand the association between the implementation of SSPs and STI, HIV, and HCV rates in Florida counties, the DID method was used. DID is a quasi-experimental design that allows for the comparison of the change in STI, HIV, and HCV rates over time between counties that implemented SSPs (treatment group) and those that did not (control group). DID is widely used in economics and policy research and is an effective technique for assessing the effects of public health interventions and policies in population health and epidemiologic research.^22^^,^^23^ Analyses were conducted in Stata 18 to carry out a 2-way fixed effects DID model using panel data to estimate the average treatment effect on the treated.^24^ DID assumes parallel trends, meaning that in the absence of treatment (i.e., SSP implementation), the rates of STIs, HIV, and HCV in the treatment and control counties would remain constant, so any differences in rates can be ascribed to treatment rather than potential confounding factors. The Granger causality test is frequently assessed to examine the violation of this assumption. However, the Stata procedure used in this analysis (xtdidregress) does not perform well when the number of clusters is small. To address this limitation when comparing Miami-Dade county with other individual counties, a wild cluster bootstrap was applied to allow for the calculation of more reliable CIs, using Stata’s default settings of Rademacher weight distribution with 999 replications.^25^ SEs were clustered at the county level and included 8 selected counties in the analytic sample, corresponding to 8 clusters. This method does not estimate a variance–covariance matrix and therefore was unable to perform the formal statistical tests to assess the parallel trends assumption. Instead, the Stata estat trendsplots procedure was used to assess whether the parallel trends assumptions were violated, and visual inspection was used, which has been used in prior research.^26^ Granger causality test results are noted in the tables for completeness but were not emphasized in the interpretation; given the methodologic limitations and inconsistent applicability across models using wild bootstrap inference, the primary inference is based on the DID estimates and parallel trends assessment.

## RESULTS

Table 1 displays the rates of combined STIs (chlamydia, gonorrhea, and infectious primary and secondary syphilis), each STI individually, HCV, and HIV per 100,000 residents in the selected treatment and control counties for the years 2010 to 2023. As was observed nationally,^27^ the rates of these sexually transmitted and blood-borne infections increased during this period in Florida counties.

**Table 1:** Rates per 100,000 of Combined STIs, Chlamydia, Gonorrhea, Infectious Syphilis, HCV, and HIV in Florida Counties in 2010–2023

County	2010	2011	2012	2013	2014	2015	2016	2017	2018	2019	2020	2021	2022	2023
Combined STI rates														
Miami-Dade	493.4	490.3	524.0	550.5	567.5	624.9	669.5	660.6	733.2	811.4	721.8	853.1	910.5	1,011.7
Broward	565.8	573.3	595.7	639.3	694.3	749.3	800.3	883.1	890.2	974.3	858.1	921.6	996.0	1,156.1
Palm Beach	352.0	360.0	369.9	435.1	453.8	504.4	509.0	546.9	552.7	572.8	530.3	581.3	635.1	663.1
Bay	552.2	456.6	458.5	434.7	506.8	657.4	600.1	664.4	605.9	619.2	594.5	701.2	709.7	688.1
Collier	239.7	252.9	276.6	272.8	271.1	300.1	349.7	355.1	424.5	364.6	342.1	375.2	398.5	388.7
Pasco	246.8	278.0	265.5	288.9	273.5	340.8	367.5	391.3	412.2	447.9	452.6	431.4	386.6	402.0
Polk	505.9	488.8	489.7	554.4	584.7	644.3	638.8	668.4	681.8	786.2	750.3	759.3	729.9	776.2
St. Lucie	466.5	461.9	425.3	448.5	439.7	459.8	472.9	510.1	562.8	638.0	533.8	513.7	523.6	563.8
Chlamydia rates														
Miami-Dade	346.5	349.9	376.9	399.2	404.9	445.1	467.7	445.3	477.7	520.6	433.8	493.5	514.4	569.8
Broward	397.8	403.8	417.8	453.9	492.9	520.8	533.5	598.7	595.0	636.6	518.1	520.7	584.0	690.1
Palm Beach	264.0	274.4	294.7	335.1	350.3	388.9	391.0	417.0	417.8	434.9	385.1	401.7	434.8	457.0
Bay	432.7	386.3	382.9	376.5	405.2	491.3	444.4	458.2	397.3	415.8	415.9	463.7	482.9	465.7
Collier	209.0	223.3	243.7	239.3	239.2	258.4	305.9	301.5	352.4	297.9	261.3	280.7	298.8	294.6
Pasco	203.0	224.3	217.6	243.0	223.8	261.2	279.7	291.9	314.1	328.0	323.3	297.2	268.9	282.6
Polk	406.2	401.7	404.3	446.3	462.4	482.4	465.1	492.2	521.8	565.4	510.6	504.8	486.9	539.2
St. Lucie	371.9	348.3	342.2	343.0	344.2	377.8	374.0	392.7	425.9	449.1	381.8	351.4	355.7	394.8
Gonorrhea rates														
Miami-Dade	97.9	93.2	94.2	93.1	93.1	97.5	112.9	128.4	153.6	176.6	179.3	219.9	231.3	272.8
Broward	122.8	121.4	122.5	132.2	140.9	165.9	195.1	208.6	202.2	231.8	230.3	256.6	264.9	340.6
Palm Beach	69.4	64.9	54.8	78.7	81.3	92.4	92.3	97.6	101.4	102.6	108.0	134.9	137.4	144.4
Bay	116.6	67.3	69.0	55.9	95.2	155.2	144.9	179.9	185.5	175.1	151.3	191.1	179.8	164.4
Collier	22.0	24.6	25.4	20.3	18.3	26.0	28.1	39.3	56.9	47.9	63.9	71.7	68.6	53.1
Pasco	40.0	49.0	41.5	40.4	41.1	68.4	75.9	88.3	83.7	97.7	108.2	106.7	88.3	89.7
Polk	83.0	71.6	71.0	96.2	104.6	140.2	152.5	153.6	127.2	186.1	205.2	207.1	191.9	182.0
St. Lucie	83.7	105.7	73.1	91.7	82.4	68.8	87.0	85.0	98.4	148.8	125.4	123.2	116.7	124.9
Infectious syphilis rates														
Miami-Dade	14.9	13.0	13.6	16.0	16.4	18.5	19.8	17.4	19.4	20.7	21.9	25.0	27.0	27.2
Broward	12.5	13.4	15.9	14.8	17.6	15.9	15.9	17.5	23.4	22.4	24.6	28.7	25.6	22.9
Palm Beach	4.5	4.9	5.9	6.2	4.6	7.4	6.7	7.8	6.2	8.4	8.2	10.3	13.3	11.8
Bay	0.6	0.6	2.4	—	1.2	2.3	2.8	9.5	5.5	8.3	10.2	23.2	12.2	17.0
Collier	1.2	0.9	2.7	3.6	5.3	4.9	4.3	5.3	3.8	7.4	4.1	9.5	9.5	13.0
Pasco	1.3	1.3	2.6	2.3	3.9	3.9	3.6	1.8	3.3	7.4	6.7	6.6	9.5	10.3
Polk	7.5	7.1	6.7	5.5	7.8	8.6	7.8	5.6	10.0	12.2	12.3	16.9	19.2	16.4
St. Lucie	2.9	3.2	1.4	2.8	3.2	1.4	2.7	10.0	12.5	12.3	9.8	13.1	15.4	9.1
HCV rates														
Miami-Dade	0.0	0.0	0.0	0.0	0.0	0.0	0.1	0.6	2.2	1.6	6.5	8.3	10.2	10.2
Broward	0.4	0.2	0.5	0.8	0.4	0.4	1.3	1.4	2.6	3.0	5.9	5.8	4.7	6.9
Palm Beach	0.2	0.8	0.8	1.2	0.7	0.7	0.7	2.7	2.7	2.0	7.6	7.0	6.3	5.5
Bay	1.2	0.6	0.6	1.2	0.6	0.0	1.1	1.7	2.7	1.7	4.0	6.8	4.4	5.9
Collier	1.2	1.2	2.1	2.4	1.2	1.2	2.6	3.1	1.9	2.4	5.7	7.0	3.3	4.5
Pasco	1.7	1.1	0.4	0.2	1.5	1.0	5.8	4.3	4.0	10.4	15.6	14.0	8.4	7.4
Polk	0.7	0.2	1.2	1.3	1.1	1.6	1.1	1.4	1.5	3.0	6.9	6.1	4.8	4.7
St. Lucie	2.2	2.9	2.5	2.8	2.1	2.8	3.7	6.3	4.6	6.8	12.0	9.2	4.8	7.2
HIV rates														
Miami-Dade	46.7	44.6	42.6	42.9	43.3	48.5	44.8	39.3	38.6	37.2	25.5	30.4	36.5	37.6
Broward	44.7	44.3	38.4	36.5	36.7	32.9	35.8	35.3	30.5	29.1	21.9	28.1	29.7	29.6
Palm Beach	23.6	23.4	19.4	23.3	23.1	19.9	20.4	20.4	18.5	15.4	13.6	18.6	19.3	18.3
Bay	14.8	10.0	10.0	16.5	12.8	9.8	10.2	17.3	17.6	17.8	7.4	24.3	22.1	28.2
Collier	12.7	12.6	11.2	9.9	9.5	12.7	14.2	12.0	9.8	9.3	3.6	8.0	14.8	16.0
Pasco	6.4	9.9	8.7	5.5	7.1	8.8	9.2	7.7	9.8	8.2	7.0	8.4	13.3	10.3
Polk	16.6	15.4	13.8	14.1	13.8	15.4	18.1	14.3	15.4	18.3	10.6	16.8	15.9	20.0
St. Lucie	21.2	18.9	14.6	15.3	16.9	11.8	17.0	21.7	16.1	14.2	8.5	12.2	16.5	16.9

HCV, hepatitis C virus; STI, sexually transmitted infection.

When comparing changes in STIs, HIV, and HCV rates before SSP and after SSP implementation in Miami-Dade county with those in non-SSP counties (Table 2), rates of combined STIs per 100,000 were significantly higher in Miami-Dade county than in Bay county (123.6, 95% CI=114.2, 133.1), Collier county (164.3, 95% CI=25.7, 293.0), and St. Lucie county (158.7, 95% CI=53.7, 263.7); rates in other individual control counties and the combined control counties also appeared to be higher but were not statistically significant and/or violated assumptions. Results were similar when examining chlamydia, gonorrhea, and infectious syphilis separately, although most findings were not statistically significant and did not satisfy the parallel trends assumption. When examining changes in HCV rates, all comparisons of Miami-Dade county with individual non-SSP counties violated the parallel trends assumption; however, the comparison with the combined non-SSP counties did not violate this assumption, although it showed no significant change in HCV rates. Conversely, except for comparisons with Collier county, rates of HIV in Miami-Dade county were significantly lower after SSP implementation than in all non-SSP counties individually and combined, while satisfying assumptions. For example, HIV cases decreased by approximately 17 per 100,000 (95% CI= −25.0, −9.0) in Miami-Dade county versus Bay county before versus after SSP implementation, and compared with all non-SSP counties combined, rates decreased by approximately 11 per 100,000 (95% CI= −15.4, −6.7).

**Table 2:** Difference-in-Difference Estimates and 95% CIs of Change in STBBI Rates in Miami-Dade County Versus Control Counties Before SSP Implementation (2010–2016) and After SSP Implementation (2017–2023)

Treatment county versus control county	Combined STIs	Chlamydia	Gonorrhea	Infectious syphilis	HCV	HIV
Miami-Dade versus Bay	**123.6 (114.2, 133.1)**^a^	69.7 (−25.6, 164.9)^a^	22.7 (−3.2, 48.7)	−**4.0 (**−**6.8,** −**1.2)**	2.5 (−0.9, 5.9)	−**17.0 (**−**25.0,** −**9.0)**^a^
Miami-Dade versus Collier	**164.3 (25.7, 293.0)**^a^	42.6 (−19.4, 104.7)	63.6 (−0.1, 127.4)	2.4 (−0.7, 5.5)^a^	**3.4 (0.5, 6.2)**	−8.4 (−25.2, 8.4)^a^
Miami-Dade versus Pasco	**131.3 (64.0, 198.7)**	30.5 (−56.0, 117.0)	53.7 (−7.5, 114.9)	2.8 (−1.5, 7.2)^a^	−1.8 (−10.2, 6.5)	−**11.1 (**−**14.6,** −**7.6)**^a^
Miami-Dade versus Polk	76.7 (−172.7, 326.0)	16.3 (−17.0, 49.7)^a^	**21.1 (5.9, 39.4)**	0.7 (−1.9, 3.2)^a^	2.6 (−2.9, 8.2)	−**10.3 (**−**16.7,** −**4.0)**^a^
Miami-Dade versus St. Lucie	**158.7 (53.7, 263.7)**^a^	59.6 (−114.7, 233.8)	**64.6 (23.6, 105.6)**	−**2.6 (**−**3.5,** −**1.7)**	1.1 (−0.2, 2.4)	−**8.4 (**−**11.1,** −**5.6)**^a^
Miami-Dade versus all control counties	**129.4 (88.3, 170.4)**^b^	**43.4 (16.9, 69.9)**	**45.1 (18.4, 71.9)**^a^**^,^**^b^	−**0.1 (**−**3.7, 3.6)**	1.5 (−1.0, 4.1)^a^^,^^b^	−**11.0 (**−**15.4,** −**6.7)**^a^

*Note*: Boldface indicates statistical significance (*p*<0.05)

a Denotes satisfaction of the parallel trends assumption.

b Indicates passing of the Granger causality test. HCV, hepatitis C virus; SSP, Syringe services program; STBBI, sexually transmitted blood-borne infection; STI, sexually transmitted infection.

In analyses that combined Miami-Dade county with neighboring Broward and Palm Beach counties as the treatment counties during the shortened span of years in which all 3 counties had their own operational SSP, the results were similar to those observed in Miami-Dade county alone, although most were no longer significant (Table 3). Specifically, rates of STIs, both combined and individually, appeared higher in counties with SSPs (i.e., treatment counties) than in those without. Changes in HCV rates were negligible, and most of those results were not statistically significant and violated assumptions. Rates of HIV were significantly decreased when comparing counties with SSPs with those without. In the analysis using a delayed postimplementation period for Miami-Dade county compared with other counties (Table 4), the results were generally similar to those of the original analysis that did not include a buffer period, with some differences in the estimated effects.

**Table 3:** Difference-in-Difference Estimates and 95% CIs of Change in STBBI Rates in Miami-Dade, Broward, and Palm Beach Counties Versus Control Counties Before SSP Implementation (2018–2020) and After SSP Implementation (2021–2023)

Treatment county versus control county	Combined STIs	Chlamydia	Gonorrhea	Infectious syphilis	HCV	HIV
All treatment counties versus Bay	27.3 (−68.7, 123.2)^a^^,^^b^	−33.7 (−70.8, 3.4)	**49.6 (9.9, 89.4)**	−5.4 (−8.9, −1.9)	0.5 (−4.2, 5.3)	−**8.6 (**−**10.5,** −**6.8)**^a^
All treatment counties versus Collier	**110.0 (14.1, 206.0)**	**39.9 (2.8, 77.0)**^a^	**49.2 (9.5, 88.9)**^a^	**1.5 (**−**5.0, 2.0)**	1.8 (−2.9, 6.6)^b^	−**3.4 (**−**5.2,** −**1.5)**^a^
All treatment counties versus Pasco	**151.3 (55.4, 247.3)**	**66.3 (29.2, 103.4)**^b^	**59.1 (19.3, 98.8)**^a^	1.1 (−2.4, 4.6)^b^	3.5 (−1.2, 8.2)	−0.4 (−2.2, 1.5)^a^
All treatment counties versus Polk	**104.7 (8.8, 200.7)**	**49.7 (12.6, 86.8)**^a^	36.6 (−3.1, 76.3)	−1.9 (−5.4, 1.6)^a^^,^^b^	2.0 (−2.7, 6.8)^a^	−0.8 (−2.7, 1.0)^a^
All treatment counties versus St. Lucie	**164.9 (68.9, 260.9)**^a^**^,^**^b^	**79.0 (41.9, 116.1)**^a^**^,^**^b^	**60.0 (20.3, 99.8)**^a^	3.1 (−0.4, 6.6)	4.2 (−0.6, 8.9)	−0.3 (−2.1, 1.6)^a^^,^^b^
All treatment counties versus all control counties	**111.6 (27.5, 195.8)**^a^**^,^**^b^	40.2 (−12.9, 93.3)^a^^,^^b^	**50.9 (23.75, 78.1)**^a^**^,^**^b^	−0.9 (−5.1, 3.2)^a^^,^^b^	2.4 (−1.0, 5.8)^a^^,^^b^	−2.7 (−6.7, 1.3)^a^^,^^b^

*Note*: Boldface indicates statistical significance (*p*<0.05)

a Denotes satisfaction of the parallel trends assumption.

b Indicates passing of the Granger causality test. HCV, hepatitis C virus; SSP, Syringe services program; STBBI, sexually transmitted blood-borne infection; STI, sexually transmitted infection.

**Table 4:** Difference-in-Difference Estimates and 95% CIs of Change in STBBI Rates in Miami-Dade County Versus Control Counties Before SSP Implementation (2010–2015) and After SSP Implementation (2018–2023)

Treatment county versus control county	Combined STI	Chlamydia	Gonorrhea	Infectious syphilis	HCV	HIV
Miami-Dade versus Bay	**156.5 (136.8, 176.1)**^a^	**87.3 (67.3, 107.3)**^a^	**29.8 (19.5, 40.0)**^a^	−3.6 (−8.2, 2.1)^a^	**3.0 (1.6, 4.3)**^a^	−17.7 (−37.9, 2.4)^a^
Miami-Dade versus Collier	**194.1 (169.2, 219.0)**^a^	**52.8 (36.2, 69.3)**^a^	**73.5 (12.9, 134.1)**^a^	**3.4 (2.7, 4.0)**^a^	**3.9 (2.2, 5.6)**^a^	−**9.3 (**−**17.2,** −**1.4)**^a^
Miami-Dade versus Pasco	**158.7 (116.6, 200.7)**	**41.4 (27.2, 55.4)**^a^	**62.1 (24.3, 99.9)**^a^	**3.4 (1.2, 5.6)**^a^	−2.5 (−5.7, 0.7)^a^	−12.2 (−26.6, 2.1)^a^
Miami-Dade versus Polk	**95.9 (42.7, 149.0)**^a^	27.3 (−21.4, 76.0)^a^	**22.3 (0.2, 44.3)**^a^	0.8 (−0.6, 2.2)^a^	**3.0 (1.5, 4.6)**^a^	−11.8 (−24.8, 1.3)^a^
Miami-Dade versus St. Lucie	**192.9 (167.1, 218.6)**	**76.3 (66.9, 85.7)**	**72.4 (18.4, 126.4)**^a^	−1.4 (−3.8, 1.0)	**1.6 (0.4, 2.8)**^a^	−**8.1 (**−**14.2,** −**1.9)**^a^
Miami-Dade versus all control counties	**157.8 (110.7, 204.9)**^a^**^,^**^b^	**56.6 (26.2, 87.1)**^b^	**52.0 (22.0, 82.0)**^a^**^,^**^b^	0.7 (−2.8, 4.2)	1.8 (−1.3, 4.9)^a^^,^^b^	−**11.8 (**−**16.4,** −**7.2)**^a^

*Note*: Boldface indicates statistical significance (*p*<0.05)

a Denotes satisfaction of the parallel trends assumption.

b Indicates passing of the Granger causality test. HCV, hepatitis C virus; SSP, Syringe services program; STBBI, sexually transmitted blood-borne infection; STI, sexually transmitted infection.

Findings were relatively robust across the sensitivity analyses conducted. Similar patterns of STIs, HIV, and HCV rates were observed when Broward and Palm Beach counties only (i.e., treatment counties) were compared with the non-SSP counties (i.e., control counties) during the period in which their SSPs were operational (Appendix Table A, available online), although the results varied in statistical significance. Furthermore, in sensitivity analyses that restricted the STI, HIV, and HCV rate data in Miami-Dade county to the shortened observation periods before and after SSP implementation, the results were relatively similar to those observed for Miami-Dade county over the longer periods (Appendix Table B), including significantly decreased rates of HIV (e.g., Miami-Dade county versus all control counties: −8.6, 95% CI= −12.6, −4.7).

## DISCUSSION

This study’s findings demonstrate a significant reduction in population-level HIV rates in Florida counties where SSPs were implemented compared with those in counties where SSPs were not implemented. Specifically, HIV rates decreased by approximately 10 cases per 100,000 after SSP implementation in counties with an SSP compared with the rates in those without an SSP. Future epidemiologic, comparative effectiveness, and implementation science research is needed to understand the effects of SSPs on other STIs and blood-borne infections, including those that may not be directly attributed to receptive syringe sharing, and the mechanisms through which SSPs may impact these outcomes, such as safer sex counseling, condom distribution, and linkage to care. Taken together, the results provide compelling evidence that SSPs are beneficial in reducing HIV at the population level in Florida and support the need to expand these programs throughout the state.

This study supports the wealth of prior research highlighting the effectiveness of SSPs in reducing HIV. Systematic reviews and meta-analyses have consistently shown that SSPs can reduce HIV transmission among PWID by over 50%.^28^^,^^29^ In addition to substantial evidence that SSPs reduce individual-level HIV risk among PWID,^8^^,^^28^ simulation modeling studies predict that SSPs prevent HIV both in PWID and among people who use drugs through other routes of administration^19^ and that temporary and/or permanent disruption of SSPs may lead to rebound outbreaks.^30^^,^^31^ The results show that in Florida counties with operational SSPs, there were approximately 10 fewer cases of HIV per 100,000 residents after implementation. Future research is needed to examine the pathways through which reduced HIV risk among PWID accessing SSPs may influence population-level rates, including by reaching a priority population of PWID that may in turn reduce risk in overlapping networks and by SSPs facilitation of access to antiretrovirals for prevention (pre-exposure prophylaxis) as well as substance use disorder treatment, all of which are vital for HIV prevention across multiple, intersecting populations at risk.^28^^,^^29^

Although relatively consistent reductions in HIV rates were observed, the results for nonviral STIs and HCV were less clear, and there are likely a number of reasons for this divergence. For over a decade, the U.S. has experienced dual epidemics of increasing rates of drug use–related harms (e.g., overdose) and nonviral STIs; among people diagnosed with infectious syphilis, opioid use and injection drug use were increasingly reported.^6^ This study’s results show that the rates of STIs appeared higher in counties with SSPs than in those without, which may indicate improved detection facilitated by SSPs. Pilot and demonstration studies have shown that integrating multisite STI testing in SSPs increases case detection, but whether this ultimately translates to reduced STI incidence has yet to be examined.^32^^,^^33^ Additional research is needed to better understand how SSPs may influence nonviral STIs. Implementation strategies, such as SSP collaboration with state Departments of Health (which is mandated by Florida Statute), can make STI testing more acceptable and accessible for a community with profound distrust in the traditional healthcare system and institutions. On-site treatment may further facilitate comprehensive sexual wellness through an SSP, a trusted institution.

This study’s results regarding the rates of HCV are consistent with those of prior systematic reviews and meta-analyses.^8^^,^^34^^,^^35^ A meta-analysis among PWID globally found that SSPs were associated with over a 50% risk reduction in Europe but not in North America^34^; a simulation modeling study of PWID in Australia estimated that SSPs would effectively control HIV transmission, but HCV would remain high, driven by receptive syringe sharing.^30^ HCV is transmitted more efficiently through unsterile injection than HIV, and HCV prevalence is higher than that of HIV among PWID.^36^^,^^37^ This underscores the importance of needs-based syringe exchange to reduce the risk of reusing and resharing syringes rather than 1:1 exchange, which is the statutorily required model in Florida SSPs.^38^ Realized impacts on HCV incidence require scaled-up coverage of harm-reduction interventions,^39^ which can take time to be achieved.^40^ In addition, unique factors may affect the risk of HCV among people who use drugs. Among PWID accessing IDEA Miami, social and structural factors, such as lack of education and homelessness, were correlated with HCV infection in addition to behavioral risk factors, such as sharing and reusing syringes.^41^ This may suggest that SSPs coupled with other multilevel intervention approaches that address structural, social, and behavioral factors are needed, such as the Florida Agency for Health Care Administration’s removal of sobriety requirements for treatment, which had been a key structural barrier perpetuating HCV risk and internalized stigma. Finally, although this study did not yet observe significant population-level effects on incident HCV rates during the shortened period for which data were available, SSPs are a critical venue for offering HCV testing and treatment as prevention.^42^^,^^43^

Sensitivity analyses may offer important insights into the length of time required for population-level effects of SSPs to emerge. When the observation periods for Miami-Dade county were reduced, and the postimplementation window was limited to either fewer years in the preimplementation and postimplementation periods or with a lag between the 2 periods, previously observed significant reductions in HIV rates were attenuated and/or were no longer significant. This time lag highlights the importance of allowing for sufficient observation time to identify potential effects of SSPs at the population level and to consider the appropriate periods of observation needed. Future research should continue to explore effects as more data become available and as more Florida counties implement SSPs.

### Limitations

There are important additional limitations to consider. Although the aim of the current analysis was to explore changes in STIs, HIV, and HCV rates at the population level, the results must be interpreted considering the potential for ecologic fallacy, in which one may erroneously conclude that associations observed at the group level are also present at the individual level. Using STI, HIV, and HCV rates at the county level, one cannot draw conclusions about a causal relationship between the use of SSPs and STI, HIV, and HCV risk among individual PWID, including whether the individuals accessing SSPs in Florida are the same individuals experiencing these outcomes.^44^ Furthermore, this secondary data analysis relies on publicly available data regarding SSP implementation and STI, HIV, and HCV rates, which may be delayed and underreported. Syringe exchange may occur in the selected control counties outside of state-sanctioned SSPs, although it is very limited, and the quality of STI, HIV, and HCV surveillance rate data may vary across counties owing to variations in resources for testing and reporting. Coronavirus disease 2019 (COVID-19) disrupted STI, HIV, and HCV prevention and care, including screening; thus, the use of surveillance data from that period must be interpreted with caution.^45^ Finally, the findings may not be generalizable to other states or regions, although, unfortunately, similar to many states, particularly in the Southern U.S., Florida is both heavily impacted by HIV and lacks a robust harm-reduction infrastructure. Despite these limitations, this study is among the few to explore population-level effects of SSPs in Florida on a range of STIs and blood-borne infections, using a quasi-experimental design, thus filling an important gap and offering practical insights that may assist decision makers in implementing and refining SSP strategies in Florida and nationally.

## CONCLUSIONS

This study explores the association between implementing SSPs and population-level rates of STIs, HIV, and HCV in Florida and provides evidence to suggest that SSPs may significantly reduce HIV rates. This study contributes valuable evidence to support the expansion of SSPs, aligning with public health objectives to prevent the spread of HIV and to end the epidemic. Future research should continue to evaluate the population-level effects of SSPs on a range of STIs and blood-borne infections and on other health outcomes as well as optimal implementation approaches, such as SSP modalities, including both fixed-site and mobile SSPs, and the services offered, especially as SSPs expand, and their operational frameworks evolve.

## CRediT authorship contribution statement

**Bedis Elkamel:** Formal analysis, Data curation, Writing – review & editing. **Shelby M. Meaders:** Conceptualization, Methodology, Writing – review & editing. **Megan E. Murphy:** Conceptualization, Methodology, Writing – review & editing. **Teresa A. Chueng:** Conceptualization, Methodology, Writing – review & editing. **Hansel E. Tookes:** Conceptualization, Methodology, Writing – review & editing. **Joy D. Scheidell:** Conceptualization, Methodology, Formal analysis, Data curation, Writing – original draft, Funding acquisition, Supervision.

## Appendix Supplementary materials

Image, application 1

## Footnotes

Supplementary material associated with this article can be found in the online version at doi:10.1016/j.focus.2026.100536.
