# Supplementary material for: Exploring Sexually Transmitted and Bloodborne Infection Rates in Florida After Implementation of Syringe Services Programs: A Quasi-Experimental Study
Source: AJPM Focus. 2026 Jul 22;5(6):100536. doi: 10.1016/j.focus.2026.100536 (PMC13634733; doi:10.1016/j.focus.2026.100536)
Supplement: Supplementary file 1 [file mmc1.pdf]

Appendix

Exploring sexually transmitted and bloodborne infection rates in Florida after implementation of syringe services programs: A quasi-experimental study

AJPM Focus

Bedis Elkamel, Shelby M Meaders, Megan E Murphy, Teresa A Chueng, Hansel E Tookes, Joy D Scheidell

Table A. Difference-in-difference estimates and 95% confidence intervals of change in STBBI rates in Broward and Palm Beach Counties versus Control Counties pre-SSP implementation (2018-2020) and post-SSP implementation (2021-2023)

Treatment County vs Control County	*Combined STIs*	*Chlamydia*	*Gonorrhea*	*Infectious Syphilis*	*HCV*	*HIV*
Broward and Palm Beach Counties vs Bay	**2.7 (-95.7, 101.0)** †‡	**-44.3 (-52.5, -36.1)** †‡	42.6 (-29.3, 114.5) †‡	**-6.2 (-10.7, -1.8)** ‡	**-0.8 (-1.3, -0.4)** †‡	**-8.1 (-10.3, -6.0)** †‡
Broward and Palm Beach Counties vs Collier	85.4 (-13.0, 183.8) ‡	**29.3 (21.1, 37.5)** †‡	42.2 (-29.7, 114.1) †‡	-2.3 (-6.8, 2.1) †‡	**0.5 (0.01, 0.92)** †‡	**-2.9 (-5.1, -0.8)** †‡
Broward and Palm Beach Counties vs Pasco	**126.7 (28.3, 225.1)** ‡	**55.7 (47.5, 63.9)** †‡	52.1 (-19.8, 123.9) †‡	0.2 (-4.2, 4.7) †‡	**2.1(1.7, 2.6)** ‡	0.1 (-2.1, 2.3) †‡
Broward and Palm Beach Counties vs Polk	80.1 (-18.3, 178.5) ‡	**39.1 (30.9, 47.3)** †‡	29.6 (-42.3, 101.5) ‡	-2.8 (-7.2, 1.7) †‡	**0.7 (0.2, 1.1)** †‡	-0.4 (-2.5, 1.8) †‡
Broward and Palm Beach vs St. Lucie	**140.3 (41.9, 238.7)** †‡	**68.4 (60.2, 76.6)** †‡	53.0 (-18.9, 124.9) †‡	2.2 (-2.2, 6.7) ‡	**2.8 (2.3, 3.3)** †‡	0.2 (-2.0, 2.3) †‡
Broward and Palm Beach vs All Control Counties	**87.0 (12.0, 162.1)** †‡	29.6 (-20.8, 80.1) †‡	**43.9 (10.7, 77.1)** †‡	-1.8 (-5.9, 2.4) †‡	1.0 (-0.6, 2.7) †‡	-2.2 (-6.4, 1.9) †‡

† denotes satisfaction of the parallel trends assumption
‡ indicates passing of the Granger causality test
Bolded estimate denote statistical significance (p<0.05)

Table B. Difference-in-difference estimates and 95% confidence intervals of change in STBBI rates in Miami-Dade County versus Control Counties pre-SSP implementation (2013-2016) and post-SSP implementation (2017-2020)

Treatment County vs Control County	*Combined STIs*	*Chlamydia*	*Gonorrhea*	*Infectious Syphilis*	*HCV*	*HIV*
Miami-Dade vs Bay	**72.7 (-157.5, 302.9)** †	65.5 (-2157.0, 2288.0)	4.0 (-17.9, 25.8)	-4.7 (-43.3, 33.9) †	-0.01 (-0.1, 0.03)	-13.8 (-80.3, 52.7) †
Miami-Dade vs Collier	40.0 (-157.9, 237.9)	-7.5 (-29.3, 14.3) †	28.5 (-188.8, 245.7) †	**0.3 (0.04, 0.5)**	0.6 (-0.6, 1.8)	-5.4 (-35.9, 25.1)
Miami-Dade vs Pasco	24.6 (-668.4, 717.5)	-14.5 (-615.0, 586.1)	24.3 (-66.8, 115.3)	**0.6 (0.04, 1.1)**	-2.0 (-5.2, 1.1)	-7.4 (-23.4, 8.7) †
Miami-Dade vs Polk	24.9 (-79.0, 128.7)	-14.5 (-121.4, 92.3)	29.2 (-30.3, 88.6)	-0.3 (-0.7, 0.2)	0.7 (-0.42, 1.9) †	-7.4 (-36.1, 21.3) †
Miami-Dade vs St. Lucie	1.6 (-14.7, 17.9)	-15.3 (-287.6, 257.1)	**21.0 (8.7, 50.8)**	-8.2 (-19.3, 2.8)	-	-9.3 (-56.6, 38.1) †
Miami-Dade vs All Control Counties	**32.8 (0.3, 65.2)**	2.7 (-40.7, 46.1) †‡	**21.4 (8.7, 34.1)** †‡	-2.5 (-7.3, 2.3)	-0.5 (-2.0, 1.1) †‡	**-8.6 (-12.6, -4.7)** †

† denotes satisfaction of the parallel trends assumption
‡ indicates passing of the Granger causality test
Bolded estimate denote statistical significance (p<0.05)
